# Insight into Surface Properties of Anodic Oxidized Ti6Al7Nb Alloy for Biomedical Applications

**DOI:** 10.3390/ma19163387

**Published:** 2026-08-10

**Authors:** Karolina Wilk, Maciej Krzywiecki, Lucyna Grządziel, Marcin Godzierz, Ada Orłowska, Sławomir Suchoń, Miłosz Chrzan, Michał Burkacki, Wojciech Kajzer, Janusz Szewczenko

**Affiliations:** 1 Department of Biomaterials and Medical Device Engineering, Faculty of Biomedical Engineering, Silesian University of Technology, 41-800 Zabrze, Poland; ada.orlowska@polsl.pl (A.O.); wojciech.kazjer@polsl.pl (W.K.); 2 Institute of Physics, Centre for Science and Education, Silesian University of Technology, 44-100 Gliwice, Poland; maciej.krzywiecki@polsl.pl; 3 Centre of Polymer and Carbon Materials, Polish Academy of Sciences, 41-819 Zabrze, Poland; mgodzierz@cmpw-pan.pl; 4 Department of Biomechatronics, Faculty of Biomedical Engineering, Silesian University of Technology, 41-800 Zabrze, Poland; slawomir.suchon@polsl.pl (S.S.); milosz.chrzan@polsl.pl (M.C.); michal.burkacki@polsl.pl (M.B.)

**Keywords:** anodic oxidation, Ti6Al7Nb titanium alloy, XPS surface chemical analysis, surface roughness, wettability, corrosion resistance, surface chemistry

## Abstract

The Ti6Al7Nb alloy is increasingly applied as a vanadium-free alternative to Ti6Al4V for biomedical implants; however, implant performance is governed predominantly by the physicochemical properties of the surface layer. In this study, anodic oxidation was employed as a controlled surface engineering process to generate a functional oxide layer on Ti6Al7Nb alloy and to modify its structural, chemical, and electrochemical characteristics. The anodically formed surface layer was comprehensively characterized in terms of surface morphology, wettability, microhardness, crystallographic phase composition, electrochemical behavior, and surface chemistry combined with depth profiling analysis. In addition, the biological response of the modified surface was assessed using in vitro cytotoxicity tests. The results confirm the formation of a stable and chemically defined oxide layer with enhanced electrochemical stability and tailored surface properties. The modified surface exhibits improved corrosion resistance, controlled physicochemical parameters, and favorable cytocompatibility. These findings demonstrate that anodic oxidation enables precise engineering of the surface layer and highlight its key role as a functional interface controlling implant–environment interactions, confirming this approach as an effective strategy for the development of advanced Ti-based biomedical implant surfaces.

## 1. Introduction

The exceptional properties of titanium alloys, which include high corrosion resistance in the tissue environment, biocompatibility, high relative strength Rm/ρ, as well as a low value of Young’s modulus, the closest of all metallic materials to Young’s modulus of bone, determine their usefulness in implant applications, particularly in orthopedics. The commonly used titanium alloys in medicine are Ti6Al4V and Ti6Al7Nb. Among these alloys, Ti6Al7Nb has attracted increasing attention as a vanadium-free alternative because of its improved biological safety and favorable mechanical properties. Recent studies have demonstrated that Ti6Al7Nb exhibits excellent corrosion resistance and mechanical performance, while appropriate surface modification is essential to further enhance its biological response and long-term clinical performance [1]. Advances in titanium alloy design and processing have further expanded their applicability in advanced engineering and biomedical applications [2].

Titanium alloys have the ability to self-passivation, which is responsible for their high corrosion resistance. It results in the spontaneous formation of a passive oxide layer with a thickness of a few nanometers, which is characterized by low electronic conductivity and consists of amorphous titanium dioxide (TiO_2_), which forms its outer part, and non-stoichiometric TiO oxides_2−x_ in the inner part [1,2,3,4]. In addition, it consists of a small volume of oxides of aluminum and vanadium or niobium, depending on the chemical composition of the alloy [5]. Their presence in the surface layer can cause adverse effects in the body, such as vanadium-induced cytotoxic reactions or bone softening, which is affected by aluminum [6,7]. To eliminate the adverse effects of vanadium, the vanadium-free Ti6Al7Nb alloy is increasingly being used.

The undesirable effects of the contribution of aluminum ions to the passive layer are one of the reasons for the widely developing field of surface modification of alloys to improve their biocompatibility, as well as their physicochemical properties. The surface treatment technologies used can be most broadly divided into mechanical, chemical and physical methods [8,9,10]. Depending on the surface modification method, the following can be improved: mechanical contact between the implant and bone tissue, abrasion resistance, corrosion resistance, biocompatibility and bioactivity [9]. Ultimately, this can contribute to the success of the treatment process. Considering the desired way in which the surface of the material interacts with the bone tissue, two distinct goals of the modifications can be distinguished: improving osteoconductivity or improving osteoinductivity [11,12].

The purpose of using the mechanical method of modification is to obtain a surface with the desired topography, which determines the adhesive properties of the layer to the substrate [9]. The required surface topography is determined primarily by the intended use of the product. For example, in the case of implants used in orthopedics, in order to allow bone tissue adhesion, it is necessary to ensure adequate surface roughness [13], conversely for implants for blood contact, where the surface should be as smooth as possible [14]. This goal can be achieved by grinding, polishing or sandblasting (abrasive blasting) [8,15]. One of the most popular methods used in biomedical engineering is sandblasting with silicon carbide or oxide particles, aluminum oxide, biphasic calcium phosphates (BCPs), and hydroxyapatite particles with calcium phosphates [9,16]. Sandblasting the surface of titanium alloys causes a change in the chemical composition of the surface layer, an increase in surface roughness and a strengthening of the surface, which affects a decrease in resistance to pitting corrosion and an increase in the amount of ions penetrating from the surface [17]. Chemical methods of surface modification are based on reactions occurring at the interface between titanium alloys and the solution [16]. They lead to improved biocompatibility, bioactivity and osteoconductivity, as well as corrosion resistance, and may remove contaminants on the surface. Depending on the medium used, treatment with acids, alkalis or hydrogen peroxide can be distinguished [9].

One of the most popular methods of surface modification is anodic oxidation. In recent years, anodic oxidation has become one of the most extensively investigated electrochemical surface modification techniques for biomedical titanium alloys because it enables precise control of oxide layer thickness, morphology, chemical composition and wettability. Consequently, anodically oxidized titanium surfaces have been widely studied for orthopedic and dental implants [18]. Such a process involves the formation of an oxide layer on the surface of the anode, which is either a metal or its alloy. The reactions occurring between the anode and cathode are initiated by the flow of current from an external power source [19]. Anodization can be carried out in three ways: under potentiostatic conditions, under galvanostatic conditions, and by a combined method [20].

Obtaining a passive layer with controlled properties is possible by controlling the process parameters, which include the chemical composition of the electrolyte, pH of the electrolyte, temperature, current voltage, current density, hydrodynamic conditions and process duration [20]. The crystallographic characteristics and defect structure of the oxide layer have also been reported to influence its physicochemical properties and long-term stability [21]. The chemical composition of the bath used determines the composition of the resulting passive layer, which allows the bioactivity to be improved, or vice versa, depending on the components selected [3]. Its thickness increases faster in acidic than in basic electrolytes [22]. After the anodic oxidation process, there is an improvement in pitting corrosion resistance. However, the fatigue strength of the material decreases [3]. The value of the voltage used during the process affects the thickness of the layer, as well as its morphology. To obtain a homogeneous layer that inherits the substrate topography, the process should be carried out at medium voltage values. In this voltage range, a color effect is observed, which depends on the thickness of the oxide layer obtained. The use of a voltage lower than 40 V leads to a layer of nanotubes, which is favorable for good adhesion of hydroxyapatite particles to the substrate. This results in a much more strongly bonded and stable layer, which can extend the life of the implant. Spark anodic oxidation and plasma electrolytic oxidation methods are also promising. When conducting the plasma process, it is possible to use a current of more than 150 V, while in the case of spark oxidation, a pulsed voltage in the range of 200–500 V is used. As a result of both processes, porous layers are obtained with pore sizes that vary depending on the conditions of the processes [3,9,23]. Recent studies have also demonstrated that anodic oxidation may significantly influence protein adsorption, early cell adhesion and corrosion behavior through modifications of the oxide layer chemistry and surface energy. Therefore, detailed characterization of the produced oxide layer remains essential for understanding the relationship between processing parameters and biological performance [24].

Although anodic oxidation of titanium alloys has been extensively investigated, most studies focus primarily on corrosion resistance or biological response. Comprehensive investigations combining detailed X-ray photoelectron spectroscopy (XPS) depth profiling with physicochemical, electrochemical, structural, and biological characterization of anodically oxidized Ti6Al7Nb alloy remain limited. Moreover, the influence of mechanical surface pretreatment preceding anodization on the final oxide layer has received considerably less attention. Therefore, the aim of the present study was not to optimize anodizing parameters but to perform a comprehensive physicochemical, electrochemical, structural and chemical characterization of the oxide layer produced under previously optimized anodizing conditions. Particular emphasis was placed on detailed XPS depth profiling combined with surface morphology, corrosion behavior and biological evaluation.

## 2. Materials and Methods

### 2.1. Material

Specimens of the Ti6Al7Nb alloy, machined from a rod and compliant with the requirements of ISO 5832-11 [25], were used in the study. The study was performed using specimens with a diameter of 25 mm and a thickness of 2 mm. The surface of the samples was subjected to sequential modification processes, including grinding, sandblasting and anodic oxidation. Grinding was performed using abrasive papers with grit sizes of 180, 300 and 500. Sandblasting was carried out with glass beads of 70–110 μm in diameter for 2 min. The specimens were ultrasonically cleaned in 96% ethanol to remove surface contaminants. The final modification step consisted of anodic oxidation was conducted in an electrolyte containing sulfuric and phosphoric acids at a voltage of 97 V for 2 min. The anodizing parameters were selected based on our previous optimization studies, which demonstrated that these conditions produce a homogeneous oxide layer approximately 200 nm thick with favorable physicochemical and electrochemical properties [26].

### 2.2. Methods

#### 2.2.1. Surface Morphology Investigation

The morphology of the samples’ surface was examined using both light microscopy and scanning electron microscopy (SEM). A Leica DVM6 digital microscope (Leica Microsystems, Wetzlar, Germany) equipped with Leica LasX 5.1.0 software was employed for optical observations, while morphology analyses were performed using a Tescan Vega 4 SEM (Tescan, Brno, Czech Republic). The SEM measurements were conducted under high-vacuum conditions using electron beam energies of 10 and 15 keV. Surface topography measurements were carried out using a Leica DCM8 3D Surface Metrology Microscope (Leica Microsystems, Wetzlar, Germany) operating in confocal mode. Green light illumination and a 20× objective were used, and each measurement covered an area of 880 µm × 660 µm. The resulting surface maps were processed and analyzed using Leica Map Premium v.10 software. The study was conducted for five samples, three measurements on each sample. The results are presented as mean ± standard deviation.

#### 2.2.2. Wettability Test

The surface wettability was evaluated by measuring the static contact angle. Measurements were performed using a Biolin Scientific Attension Theta Flex optical tensiometer (Biolin Scientific, Gothenburg, Sweden), and the acquired data were processed with OneAttension 4.0.6 software. Distilled water was used as the test liquid, with a droplet volume of 1.5 mm^3^. The contact angle was determined using the Young–Laplace fitting method. Each measurement was initiated 15 s after droplet deposition and recorded for 60 s at a sampling frequency of 1 Hz. The test was conducted at room temperature. Five samples were examined with five measurements on each sample. The results are presented as mean ± standard deviation.

#### 2.2.3. Microhardness Studies

Microhardness measurements were performed using an open-platform Micro Combi Tester (CSM Instruments, Peseux, Switzerland) equipped with a Vickers indenter. The tests were carried out under depth-controlled conditions using the instrumented indentation technique in accordance with the principles of ISO 14577 [27]. Hardness was determined by the Oliver–Pharr method from the unloading segment of the load–displacement curve.

Indentation tests were performed at penetration depths ranging from 500 to 7500 nm. During each loading cycle, both the applied load and the indenter penetration depth were continuously recorded. The loading and unloading rate corresponded to a penetration speed of 3000 nm/min, and a dwell time of 15 s was applied at the maximum penetration depth. Five indentations were performed at each penetration depth on each specimen. A total of five independent specimens were analyzed, and the results are presented as mean ± standard deviation. Consecutive indentations were positioned sufficiently far apart to avoid any interaction between adjacent plastic deformation zones. Prior to testing, the instrument was calibrated according to the manufacturer’s procedure using the standard reference material supplied with the instrument.

#### 2.2.4. X-Ray Diffraction Studies

X-Ray diffraction (XRD) studies were conducted using a Bruker D8 Advance diffractometer (Bruker AXS GmbH, Karlsruhe, Germany) equipped with a Cu–Kα radiation source (λ = 1.54 Å) operating at 40 kV and 40 mA. Data were collected at a scan rate of 0.60°/min with a step size of 0.02° over the 2θ range of 5–90°. Phase identification was performed in the DIFFRAC.EVA v3.1 software using entries from the Crystallography Open Database (COD) and the International Centre for Diffraction Data (ICDD PDF#2). Precise lattice parameters and atomic coordinates were obtained through Rietveld refinement in TOPAS 6, employing the Williamson–Hall approach. A pseudo-Voigt function was applied to model the diffraction peak profiles. The refinement quality was assessed using the R_wp_ (weighted-pattern factor), R_exp_ (expected R factor), and GOF (goodness-of-fit) metrics. The population of samples was three.

#### 2.2.5. Electrochemical Investigation

Pitting corrosion resistance of samples was assessed using potentiodynamic polarization in a naturally aerated PBS solution phosphate-buffered saline (PBS; NaCl 0.14 M, KCl 0.0027 M, PO_4_^3−^ 0.01 M per 1 dm^3^) at 37 °C. PBS was selected as the electrolyte due to its ionic composition and pH, which closely resemble the natural conditions of body fluids, enabling a reliable assessment of the electrochemical stability and corrosion behavior of the investigated surfaces under biologically relevant conditions. Fresh PBS solution was used for each independent experiment. Measurements were conducted with an Autolab PGSTAT 302N (Metrohm Autolab B.V., Utrecht, The Netherlands) potentiostat operated via Nova 2.1 software. A silver/silver chloride electrode (Ag|AgCl in 3 M KCl) was used as the reference electrode, and a platinum wire served as the counter electrode. The exposed working electrode area was 4.9 cm^2^. At the initial stage, the open-circuit potential (E_ocp_) was monitored for 1 h. Potentiodynamic polarization curves were subsequently recorded beginning from an initial potential of E_init_ = E_ocp_ − 100 mV at a scan rate of 3 mV/s. Polarization was reversed when the anodic current density reached 1 mA/cm^2^ or the potential reached 2 V. Based on the obtained curves, the corrosion potential (E_corr_) and polarization resistance (R_p_) were determined using the Stern method. The population of samples was five. The results are presented as mean ± standard deviation.

#### 2.2.6. X-Ray Photoelectron Spectroscopy Studies

For the analysis of surface chemistry, X-ray photoelectron spectroscopycombined with ion-depth profiling was employed. Measurements were performed using a PREVAC spectrometer (PREVAC Sp. z.o.o., Rogów, Poland) equipped with a monochromated Al Kα X-ray source (1486.6 eV) and a dual-beam charge neutralization system. The binding energy (BE) scale was calibrated by assigning the main C 1s peak to 284.6 eV. Depth profiling was achieved through Ar^+^ ion sputtering at a constant etching rate, enabling the acquisition of chemical composition as a function of depth. Quantification of the XPS data was carried out using CASA XPS software version 2.3.25PR1.0. Peak fitting was performed primarily with Gaussian–Lorentzian product (GL) line shapes (70% Gaussian, 30% Lorentzian) following Shirley-type background subtraction. For metallic species, asymmetric peak shapes were modeled using an LA function, adjusting the Lorentzian tail on one side of the component. The uncertainty in the binding-energy position of individual components was estimated to be 0.07 eV.

#### 2.2.7. Cytotoxicity Studies

The cytotoxicity test was performed in accordance with PN-EN ISO 10993-5 [28] and PN-EN ISO 10993-12 [29], using the SaOS-2 cell line model (Human Osteosarcoma cell line, CLS, Cell Lines Service GmbH, Eppelheim, Germany, Cat. No. 300331). An MTS test was performed to determine the amount of formazan in the cells, which only living cells have the ability to produce. Extraction from the samples was carried out for 72 h at 37 ± 1 °C in DMEM/Ham’s F12 (1:1) culture medium supplemented with 10% fetal bovine serum (FBS), using an extraction ratio of 3 cm^2^ of sample surface per 1 cm^3^ of extraction medium, in accordance with PN-EN ISO 10993-5 and PN-EN ISO 10993-12. Sodium dodecyl sulfate was used as a positive control, the negative control was culture medium with bovine fetal serum. In addition, a test was performed using the Polymerase Chain Reaction (PCR) method to identify possible infection of the cell line with mycoplasma and to optimize the number of cells used in the study.

Cell survival in the test and control groups was determined by measuring the amount of formazan formed during a 72 h incubation with titanium alloy extracts, sodium dodecyl sulfate, and fetal bovine serum, respectively. The amount of formazan was determined by absorbance studies at 490 nm using a microplate reader, Multi-Mode Microplate Reader (BioTek Instruments Inc., Winooski, VT, USA). In addition, a microscopic evaluation of the effect of anodically oxidized titanium extracts on the SaOS-2 cell line was carried out. To visualize the cytoskeleton, immunofluorescence staining was performed using anti-tubulin primary antibodies and a fluorescent secondary antibody. Cell nuclei were counterstained with Hoechst. Following 72 h incubation with the extracts at 37 °C in a humidified atmosphere containing 5% CO_2_, the cells were fixed with 4% formaldehyde, permeabilized with 0.5% NP-40, and analyzed qualitatively using a Zeiss Axio Observer.D1 fluorescence microscope (Carl Zeiss Microscopy GmbH, Jena, Germany). The study was conducted for five samples.

## 3. Results

### 3.1. Surface Observations

**Figure 1:**
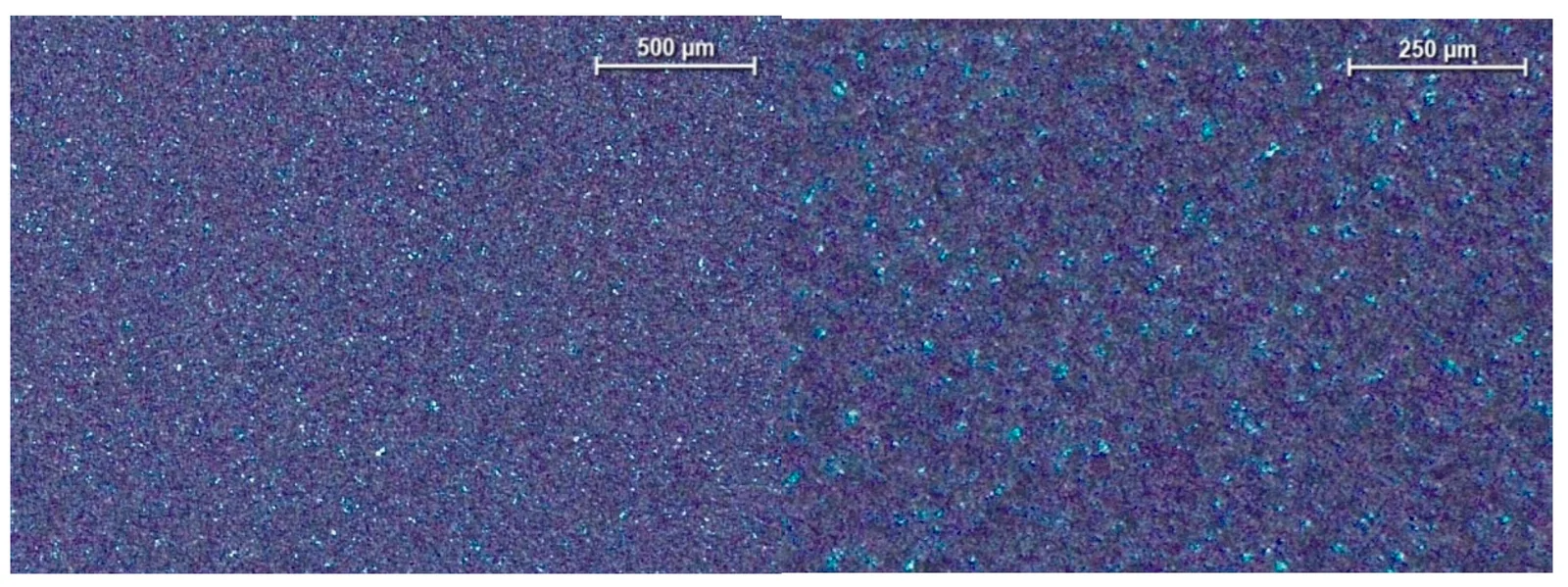
Surface of Ti6Al7Nb alloy after grinding, sandblasting and anodic oxidation observed using a light microscope. The image shows the resulting surface morphology and the characteristic coloration associated with the formation of an anodically produced oxide layer [30].

**Figure 2:**
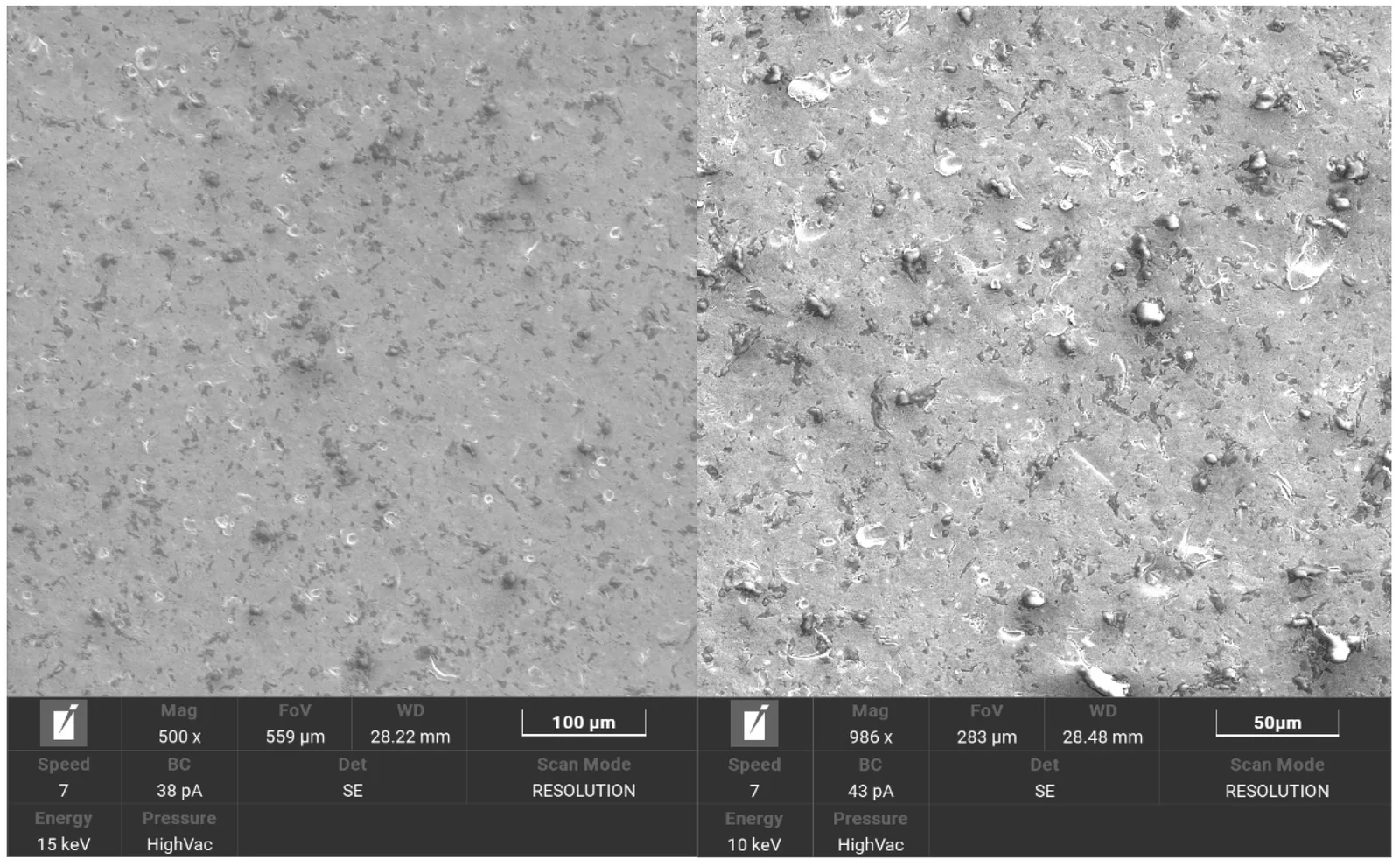
The surface of Ti6Al7Nb alloy after grinding, sandblasting and anodic oxidation, SEM. The images presents the surface topography developed during the modification process, including morphological features resulting from abrasive treatment and oxide layer formation [30].

**Figure 3:**
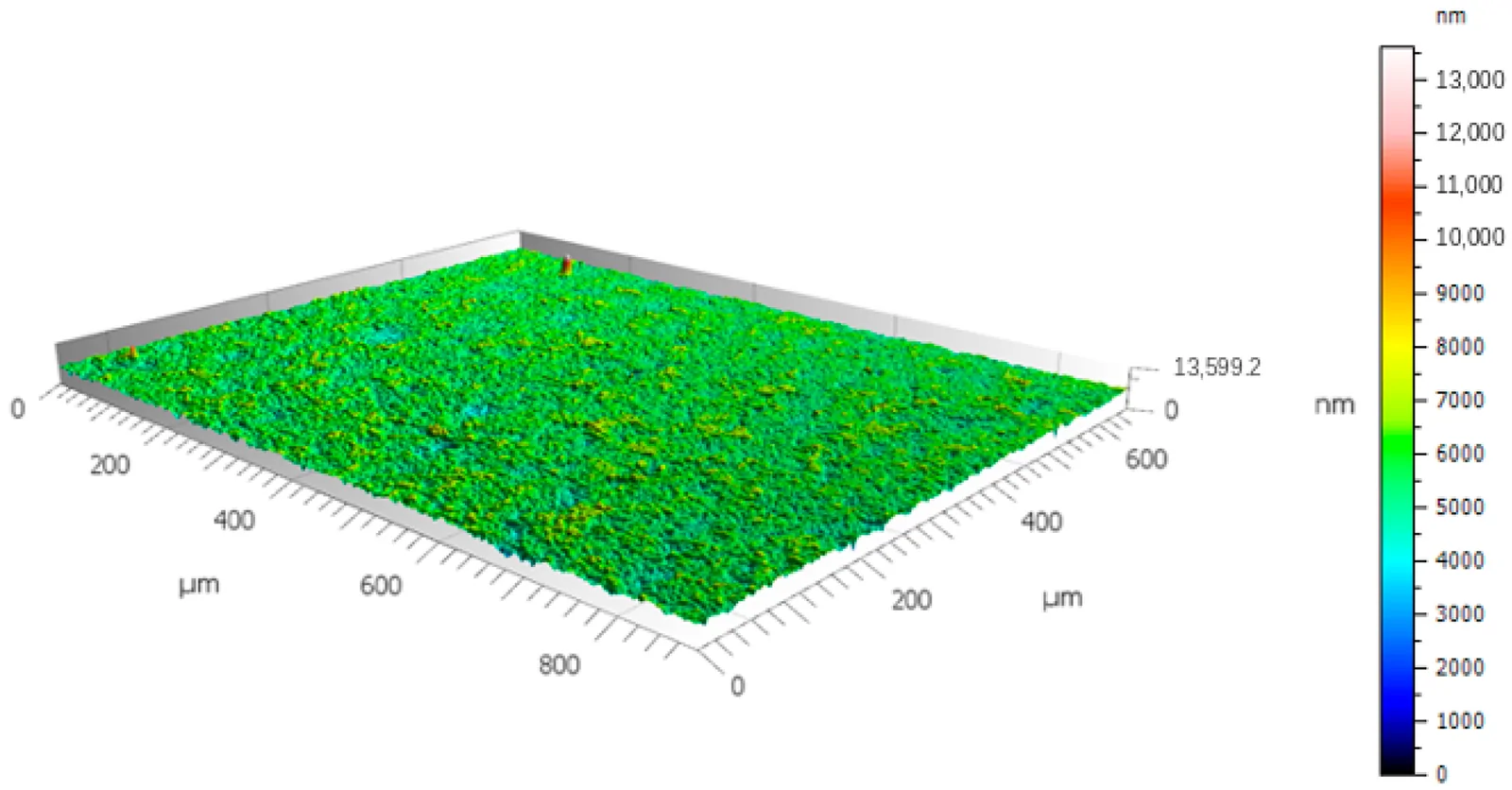
Three-dimensional surface topography of the Ti6Al7Nb alloy after sandblasting and anodic oxidation. The image illustrates the spatial distribution of surface irregularities and the morphology of the modified layer obtained after the applied surface treatments [30].

**Figure 4:**
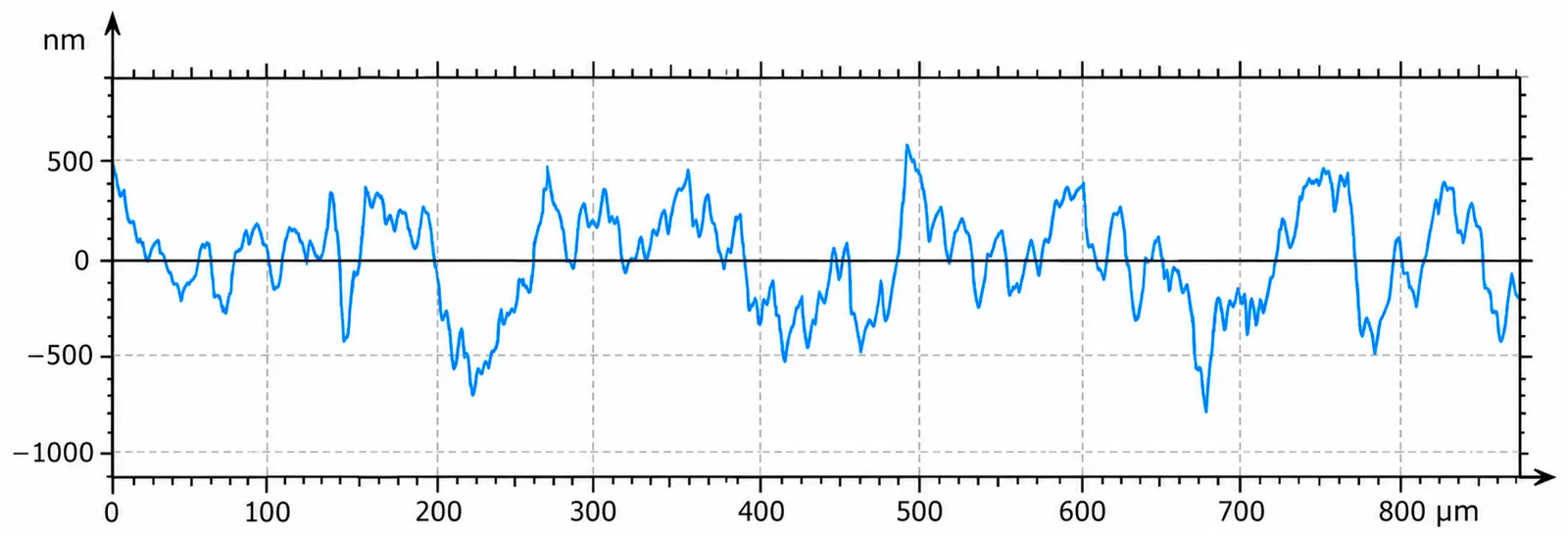
Representative surface roughness profile of the Ti6Al7Nb alloy after sandblasting and anodic oxidation. The profile demonstrates the variation in surface height and the characteristic roughness generated by the modification process [30].

The observed surface was characterized by homogeneity resulting from the sandblasting process and a color effect after anodic oxidation (Figure 1 and Figure 2). Optical microscopy observations (Figure 1) revealed a non-uniform interference color effect over the entire surface. Such color differences are characteristic of thin oxide films and result from light interference phenomena. The modified surface exhibited a homogeneous morphology with irregularly distributed asperities and valleys originating from the mechanical pretreatment (Figure 2). In addition, no evidence of localized defects or surface damage resulting from the anodizing process was observed, suggesting that the oxide layer uniformly covered the sandblasted substrate. The analysis of the obtained results made it possible to determine the surface roughness parameters as Sa = 0.396 ± 0.084 μm and Ra = 0.343 ± 0.097 μm. Examples of surface topography image and surface profile are shown in Figure 3 and Figure 4, respectively.

### 3.2. Wettability Test

**Figure 5:**
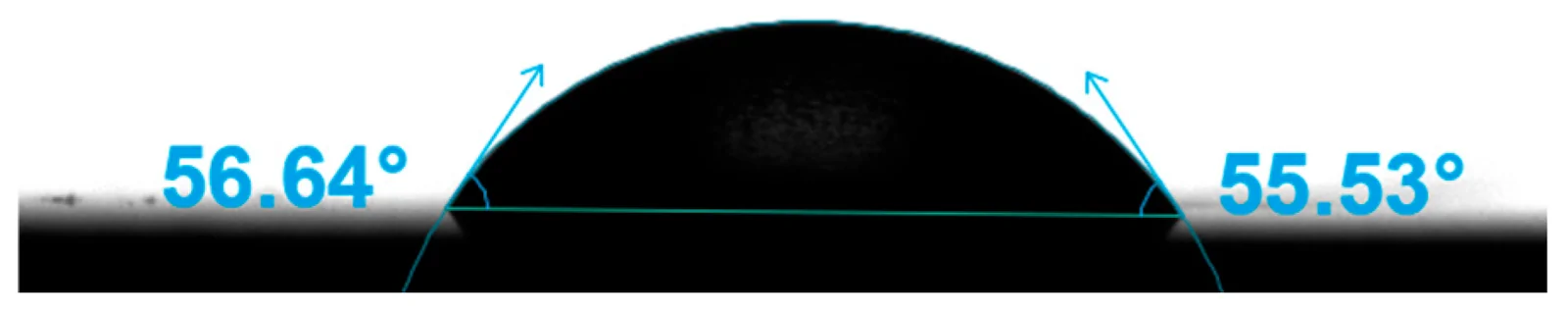
Representative water droplet deposited on the Ti6Al7Nb alloy surface after anodic oxidation. The image illustrates the wettability behavior of the modified surface used for contact angle measurement and evaluation of its hydrophilic properties [30].

The examined samples are characterized by a hydrophilic surface. The average value of the contact angle was determined as Θ = 54.7 ± 1.9°. An example of a drop on the surface of the sample is shown in Figure 5.

### 3.3. Microhardness Studies

**Figure 6:**
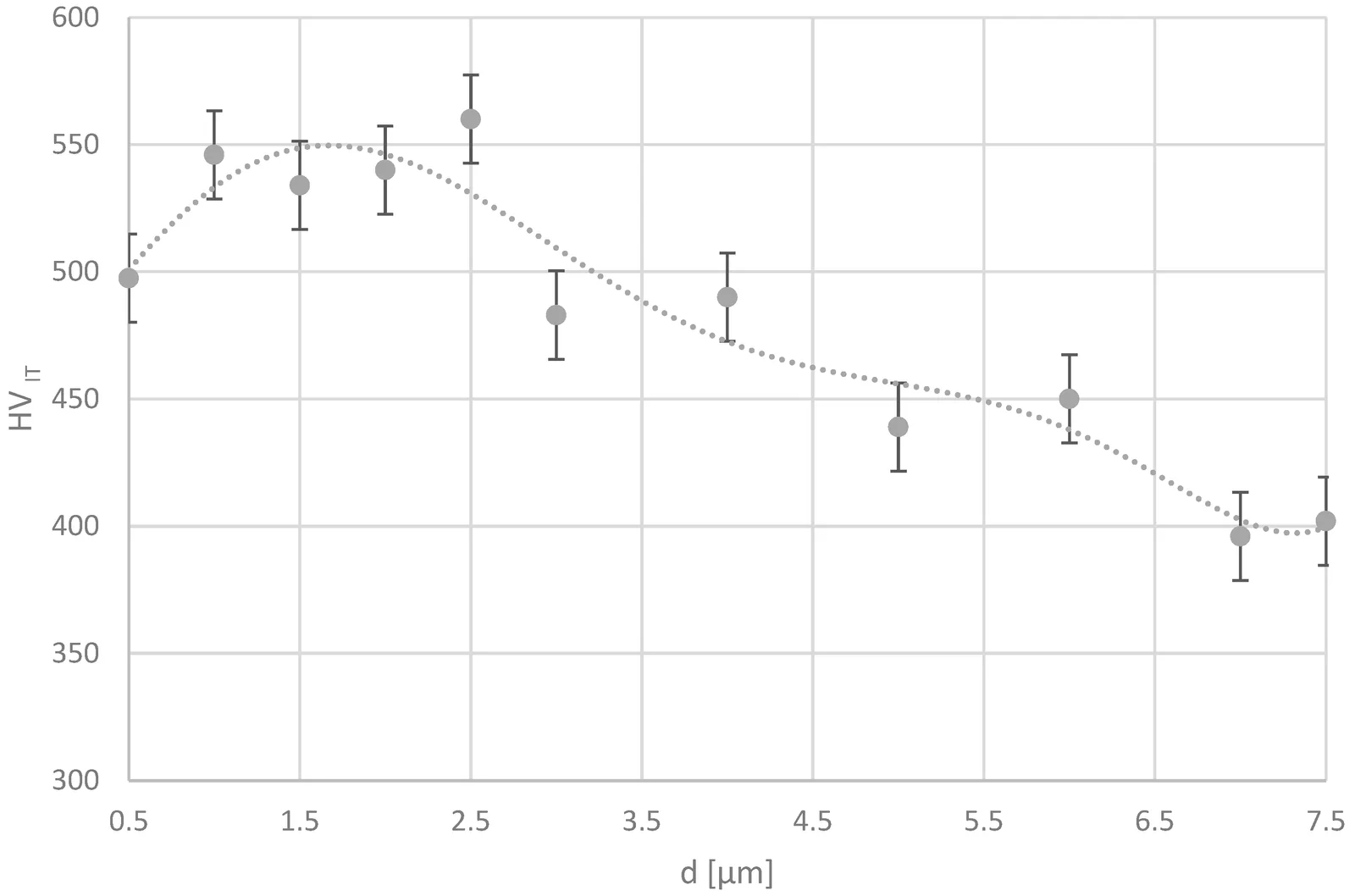
Distribution of instrumental microhardness of the modified Ti6Al7Nb alloy surface as a function of indenter penetration depth. The graph presents changes in hardness values within the surface and subsurface regions influenced by the applied modification processes [30].

The instrumental hardness of the tested surface changes with the depth of penetration. At a penetration depth of 0.5 μm, the hardness is about 500 HV, then in the depth range of 1–2.5 µm, we observe an increase in hardness, and further beyond a depth of 2.5 µm the hardness decreases until it reaches 400 HV at a depth of 7.5 µm (Figure 6).

### 3.4. XRD Studies

**Figure 7:**
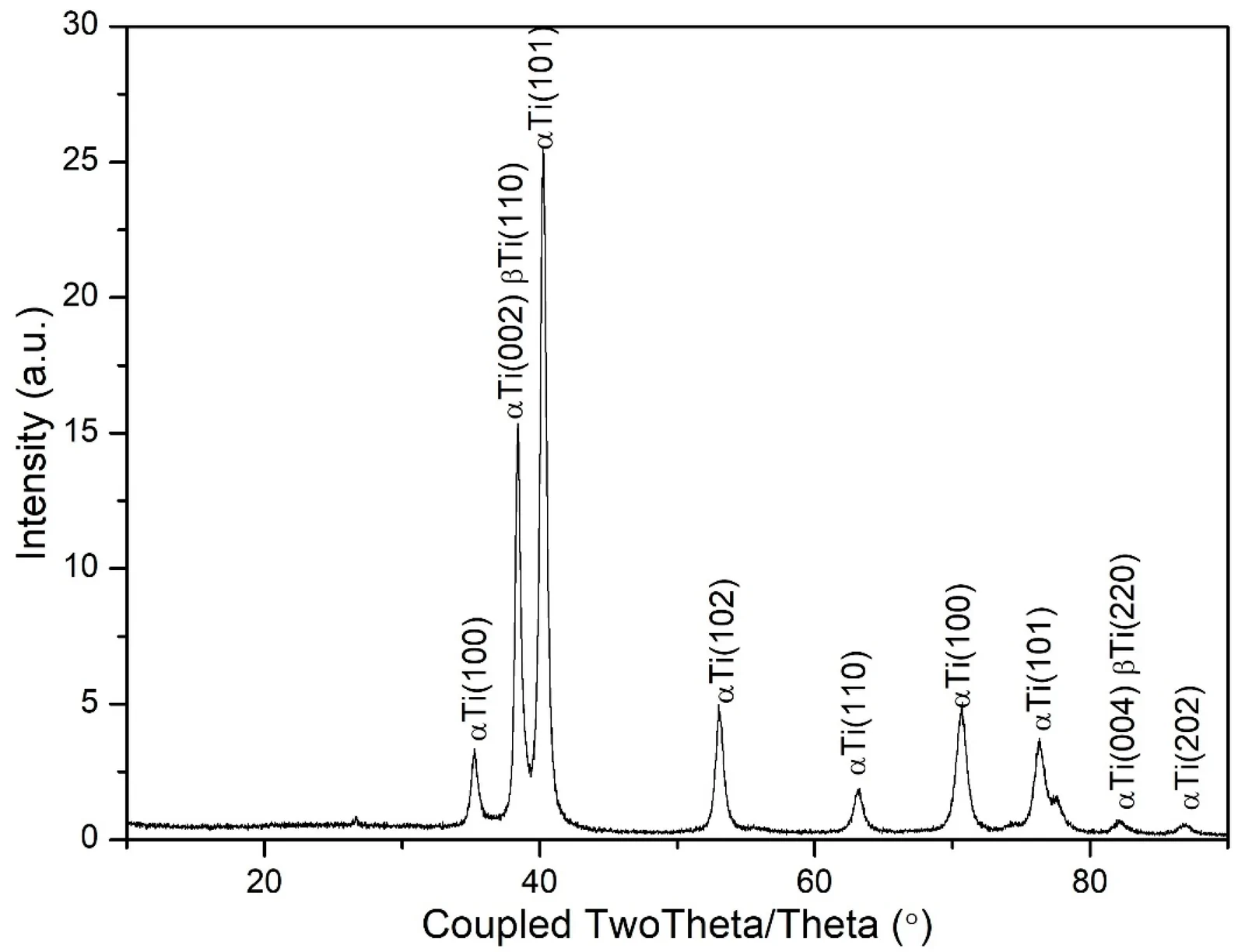
X-ray diffraction (XRD) pattern recorded for the Ti6Al7Nb alloy after surface modification. The diffractogram presents the identified crystalline phases and was used to analyze the phase composition and structural characteristics of the alloy surface layer [30].

Based on the phase analysis of the tested materials, in the surface layer αTi and βTi phases were identified (Figure 7). Selected structural parameters determined using Rietveld refinement are presented in Table 1.

### 3.5. Electrochemical Investigation

**Figure 8:**
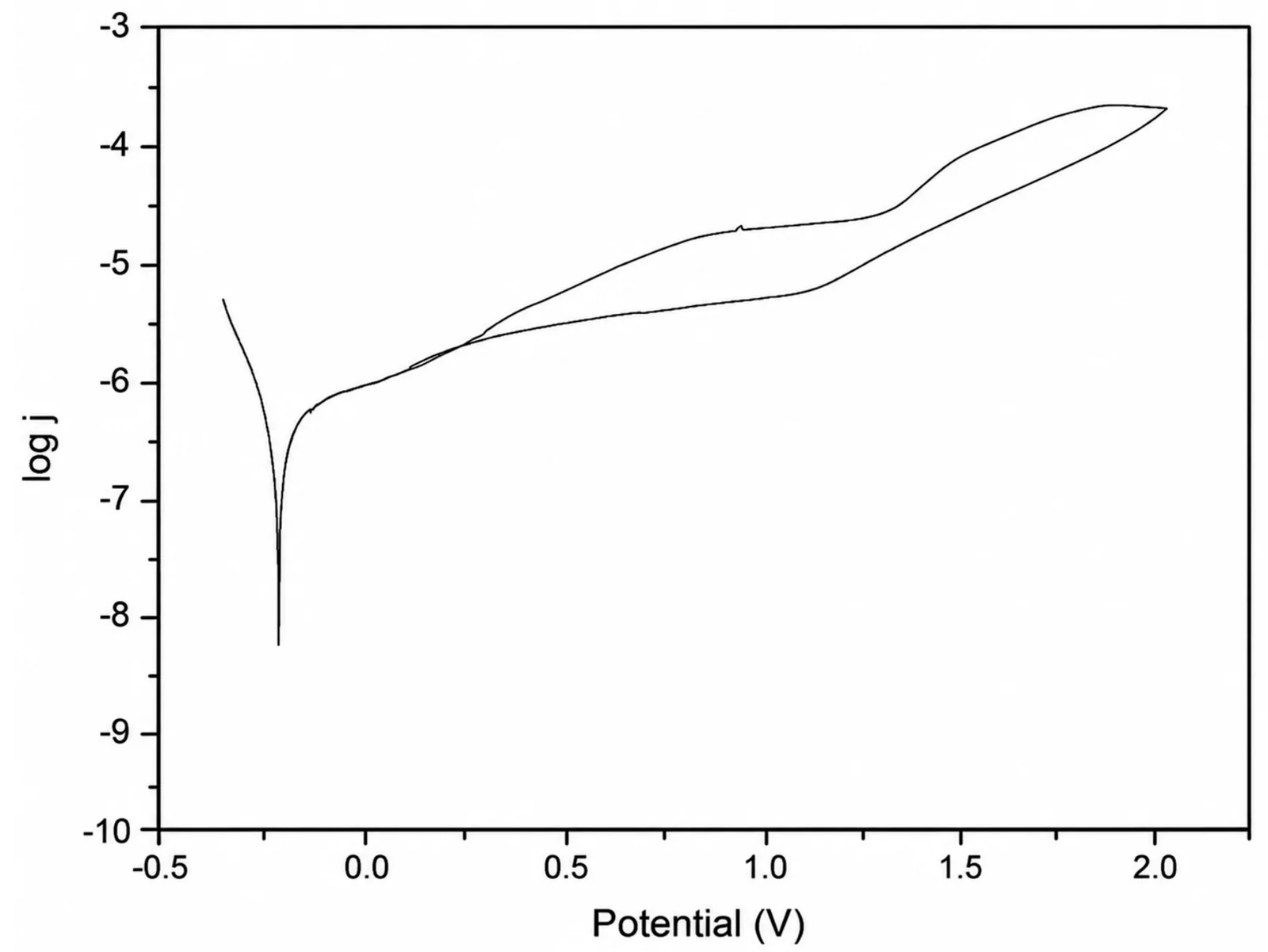
Representative potentiodynamic polarization curves obtained for the Ti6Al7Nb alloy tested in phosphate-buffered saline (PBS) solution. The curves demonstrate the electrochemical response and corrosion resistance of the anodically oxidized surface [30].

The E_ocp_ of the samples was equal to −5 ± 17 mV. For the recorded potentiodynamic curves no breakdown potential was observed (Figure 8). Based on the analysis of the recorded potentiodynamic curves, the following corrosion parameters were determined using the Stern method: corrosion potential E_corr_ = −31.2 ± 7.5 mV and polarization resistance R_p_ = 0.93 ± 0.11 MΩcm^2^. The recorded return curve is characterized by a lower current density compared to the forward curve.

### 3.6. XPS Studies

**Figure 9:**
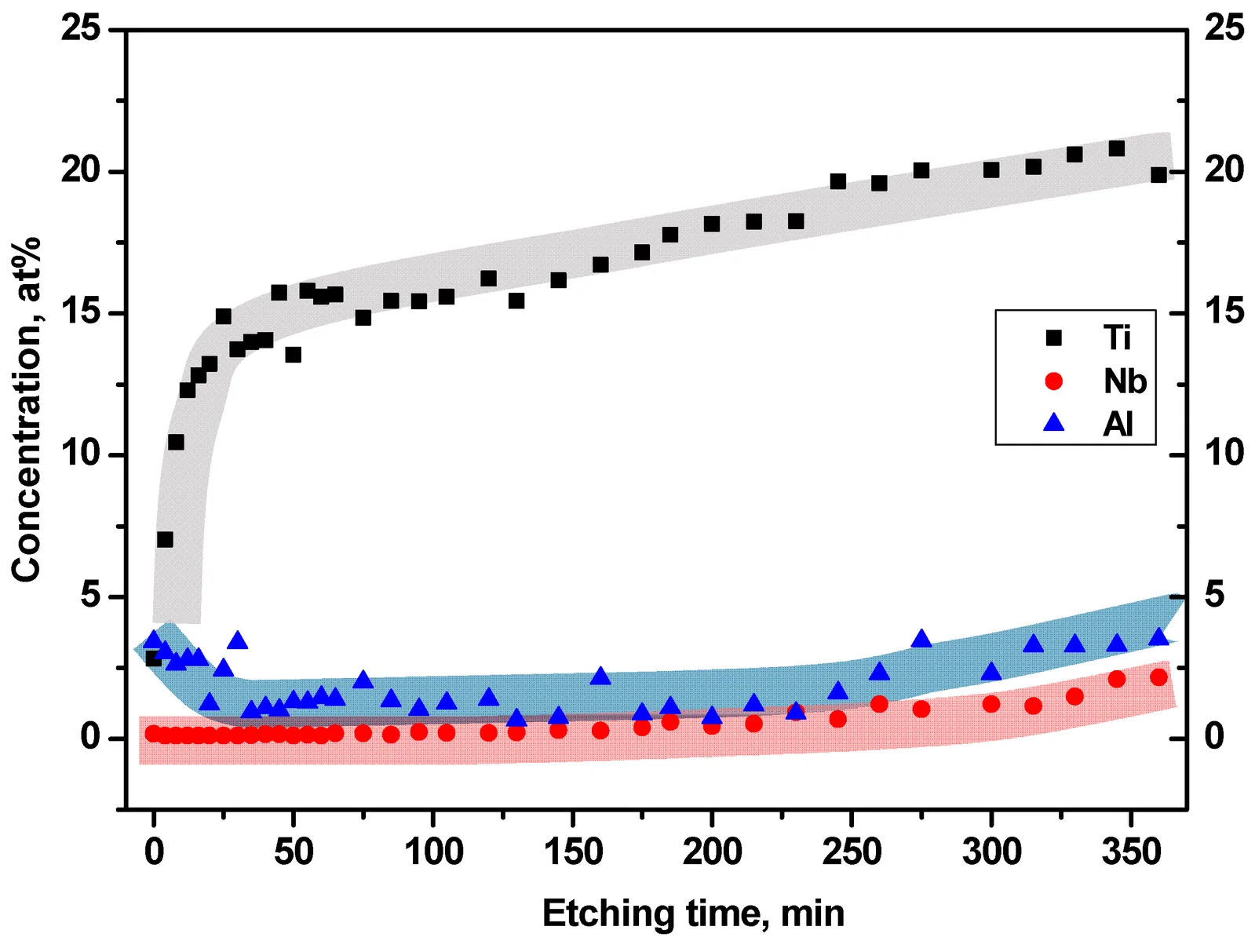
Concentration profile of Ti6Al7Nb surface as a function of Ar+ etching time for titanium, aluminum and niobium [30].

**Figure 10:**
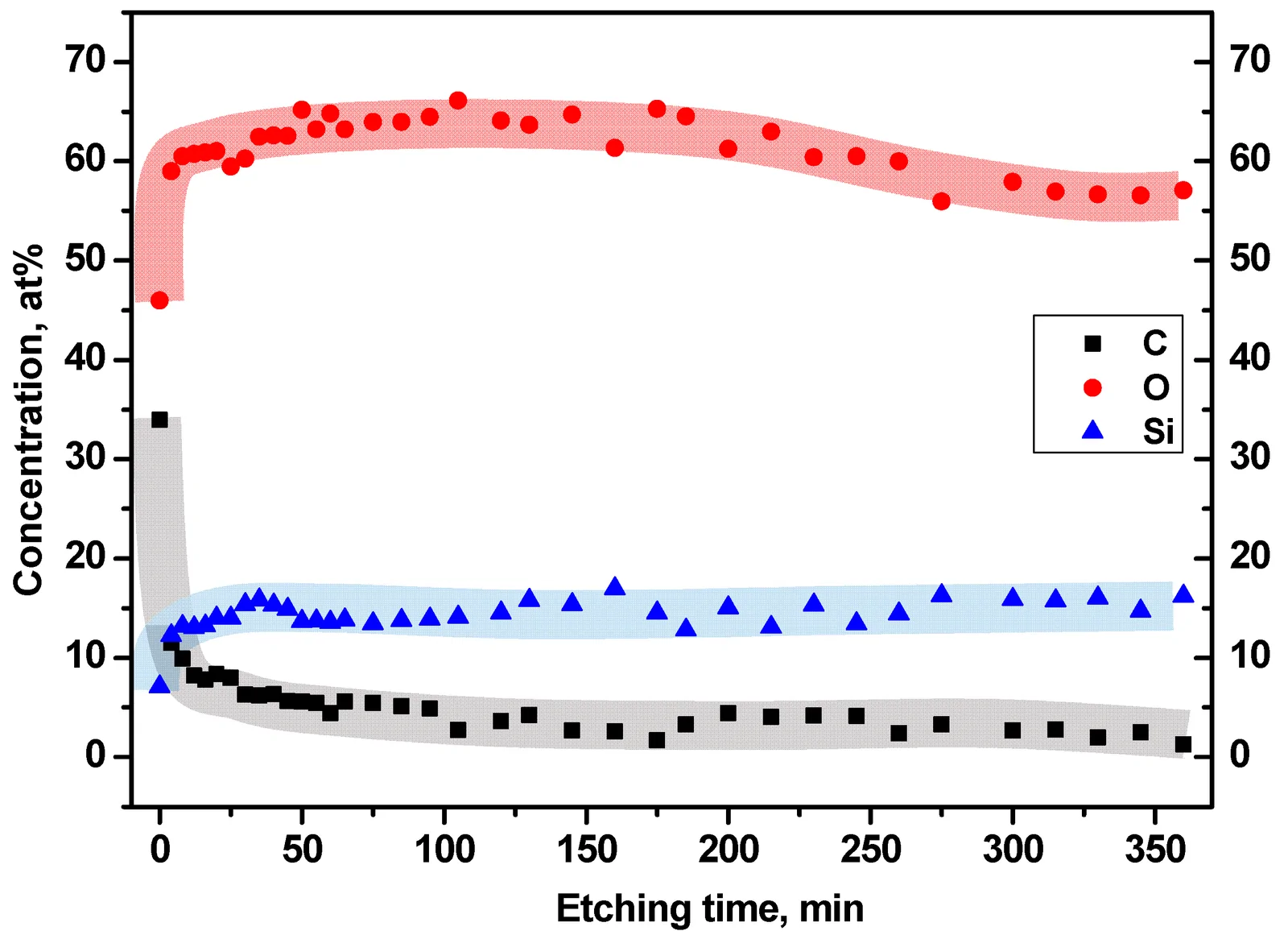
Concentration profile of Ti6Al7Nb surface as a function of Ar+ etching time for carbon, oxygen and silicon [30].

**Figure 11:**
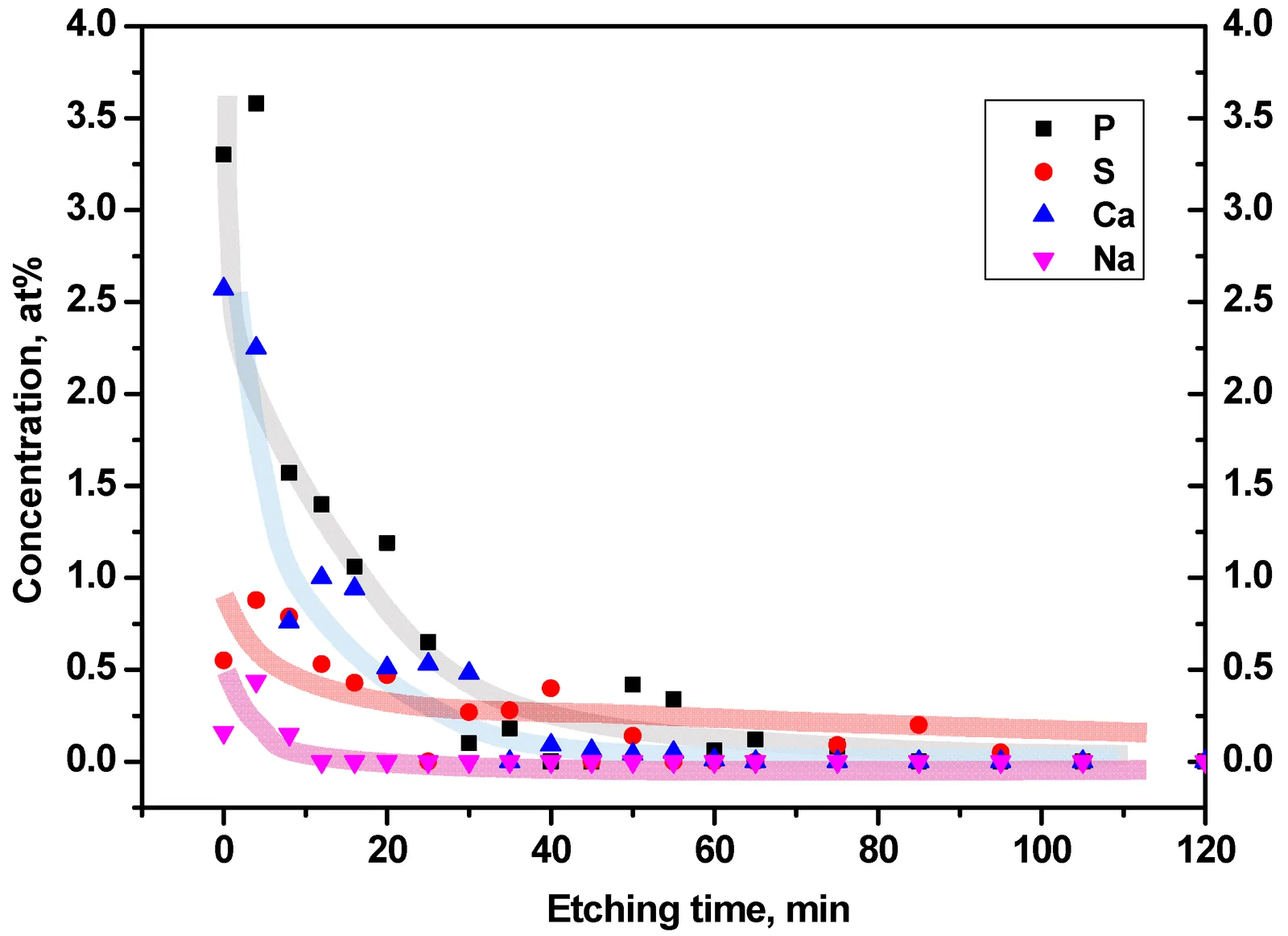
Concentration profile of Ti6Al7Nb surface as a function of Ar+ etching time for potassium, sulfur, calcium and sodium [30].

In the first step of the photoemission studies, the total concentrations of alloy constituents were investigated along the depth obtained by sequential ion etching. Figure 9 shows the percentage atomic concentration profile of the main components of the Ti6Al7Nb alloy: Ti, Al, Nb as a function of argon beam etching time. The relation was obtained based on the analysis of XPS survey spectra for individual etching times. Quantification of the spectra was based on Ti 2p, Al 2p and Nb 3d transitions taking into account the respective values of relative sensitivity factors. Figure 10 presents the percentage atomic concentration profile vs. etching time for the: O, C and Si elements that appeared in Ti6Al7Nb alloy as a result of conducted technological treatment. The dependences were obtained by quantifying XPS survey spectra for O 1s, C 1s and Si 2p transitions. Figure 11 presents the percentage atomic concentration profile vs. etching time for P, S, Ca, and Na components. They existed in Ti6Al7Nb as traces of chemical treatment on the alloy. For these sets of profile, the XPS survey spectra were quantified for P 2p, S 2p, Ca 2p and N 1s transitions.

**Figure 12:**
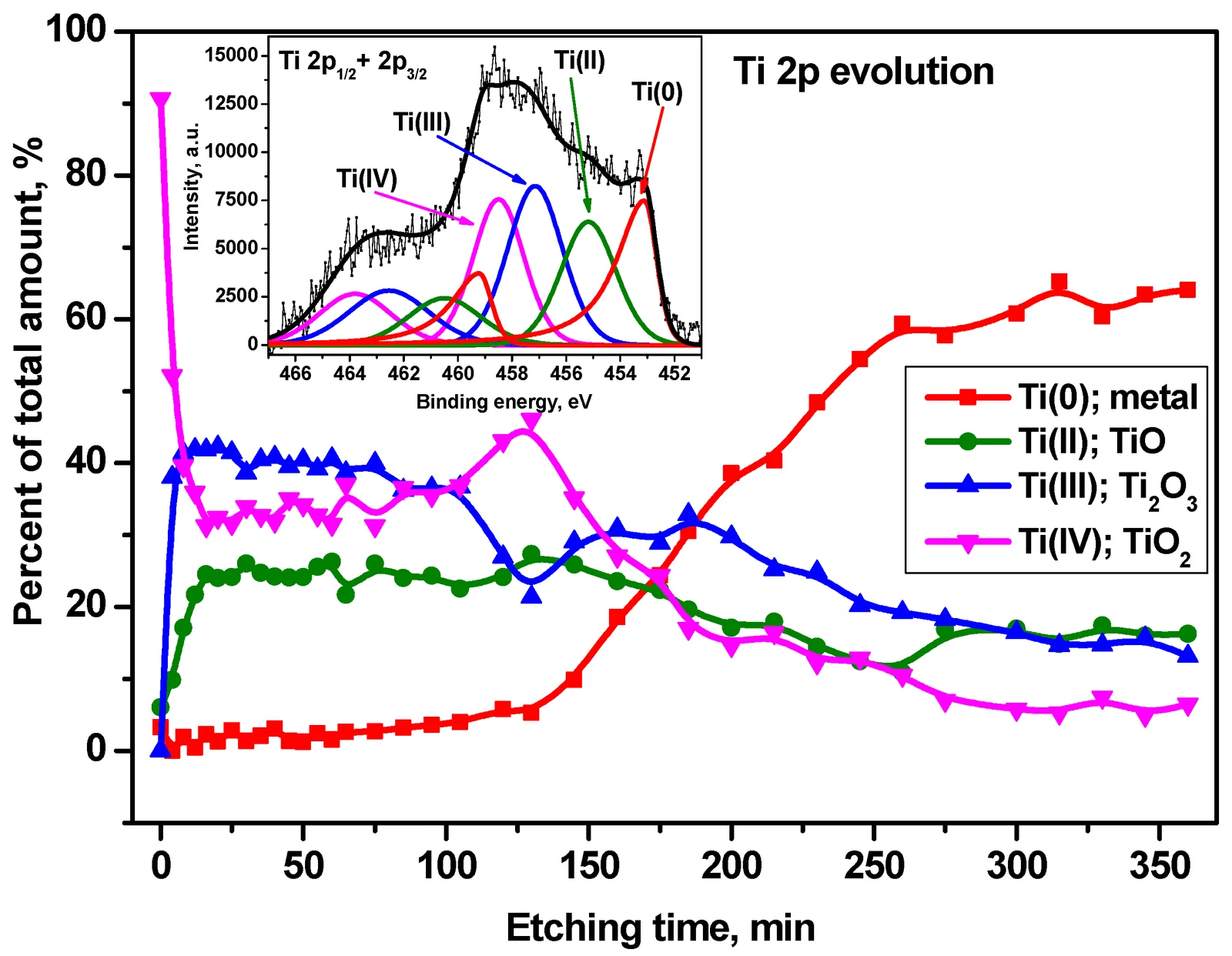
Ti 2p signal components’ evolution as a function of Ar+ etching time. Inset: XPS representative Ti 2p spectrum with peak fitting for Ti6Al7Nb surface after 175 min. of etching [30].

**Figure 13:**
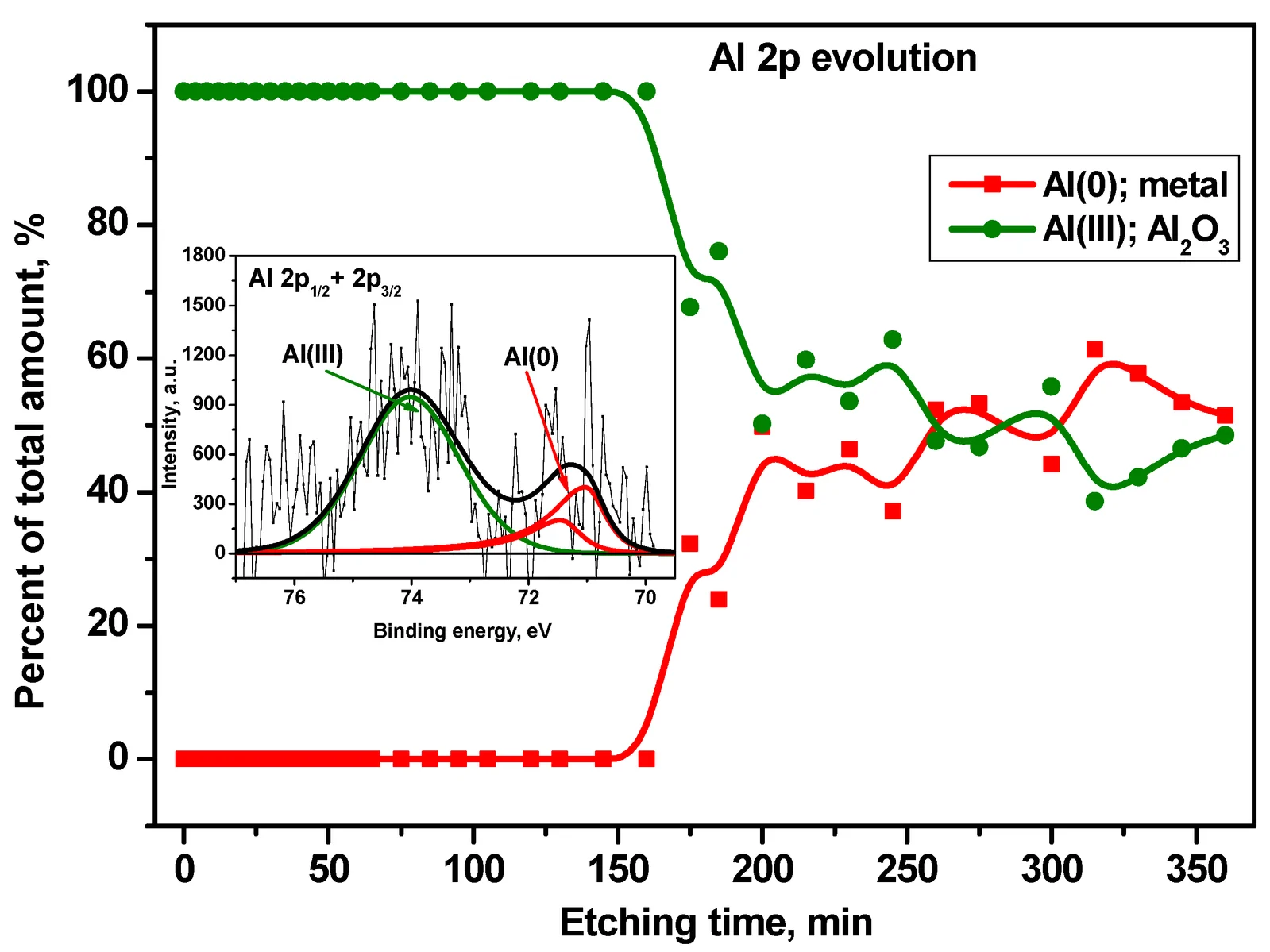
Al 2p signal components’ evolution as a function of Ar+ etching time. Inset: XPS representative Al 2p spectrum with peak fitting for Ti6Al7Nb surface after 175 min. of etching [30].

**Figure 14:**
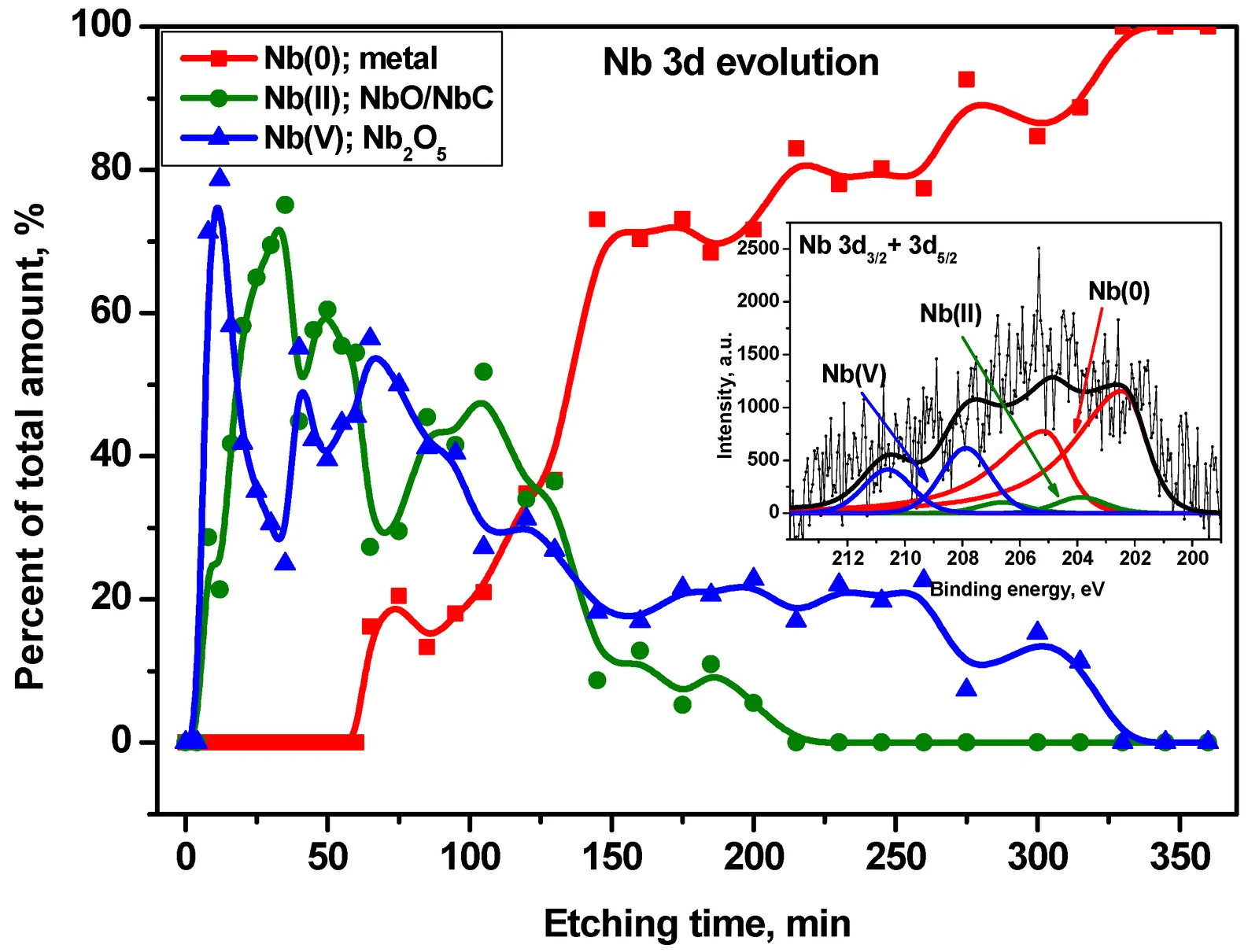
Nb 3d signal components’ evolution as a function of Ar+ etching time. Inset: XPS representative Nb 3d spectrum with peak fitting for Ti6Al7Nb surface after 175 min. of etching [30].

**Figure 15:**
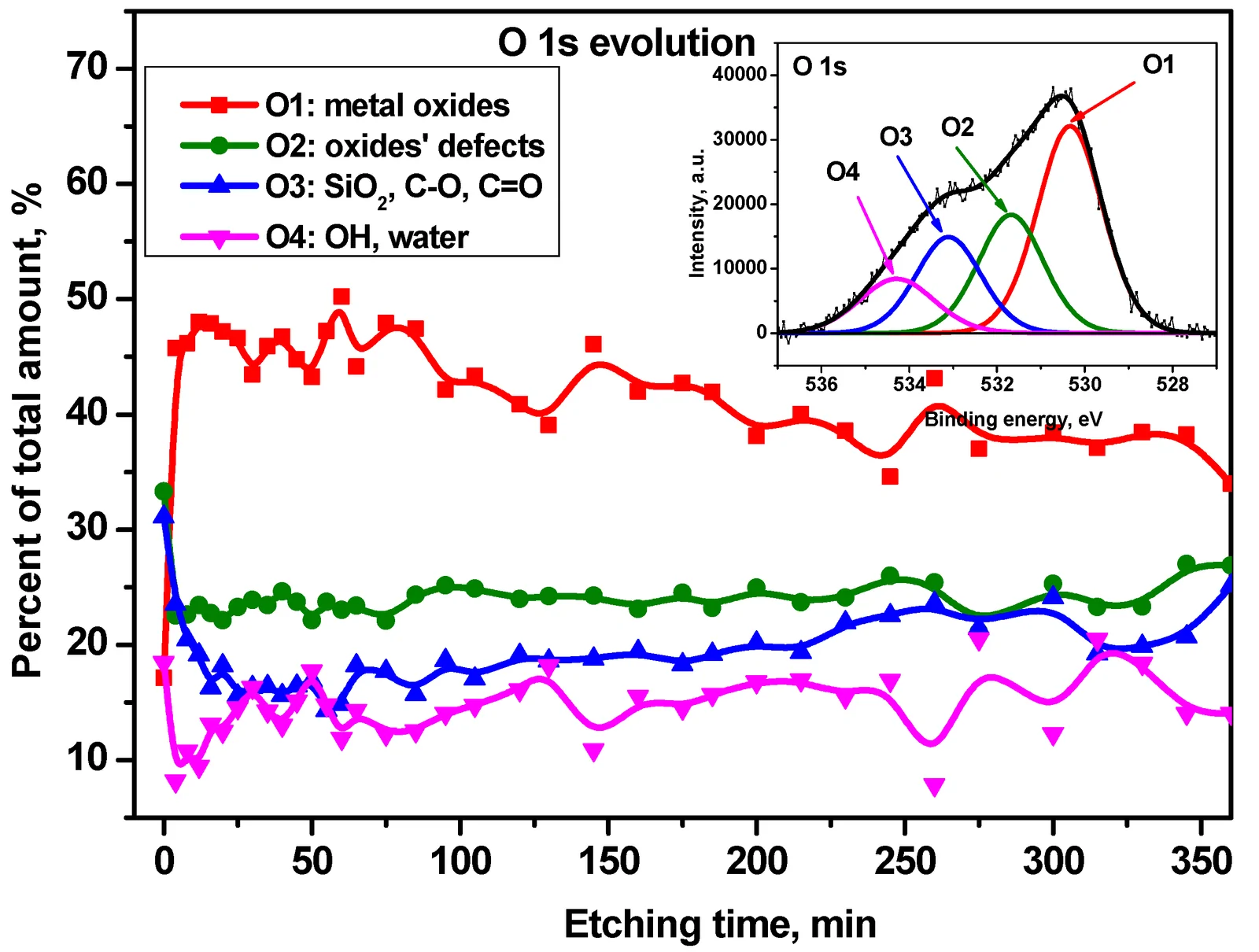
O 1s signal components’ evolution as a function of Ar+ etching time. Inset: XPS representative O 1s spectrum with peak fitting for Ti6Al7Nb surface after 175 min. of etching [30].

In the next step of the XPS examination, the chemical states of the main alloy components were analyzed. The evolution of titanium chemical states based on the Ti 2p peak components as a function of etching time is presented in Figure 12 (with exemplary window spectrum). Figure 13 presents a variation in aluminum chemical states with etching time. The analysis was conducted for the Al 2p XPS peak (exemplary window spectrum: inset to Figure 13). The evolution of niobium chemical states as a function of etching time is presented in Figure 14. The study was conducted for the Nb 3d XPS peak (exemplary window spectrum: inset to Figure 14). Figure 15 shows the evolution of oxygen chemical states based on the O 1s peak composition with etching time.

### 3.7. Cytotoxicity Studies

Taking into account the cell survival criterion, it was found that the surface of the Ti6Al7Nb alloy does not have a cytotoxic effect. The normalized survival of SaOS-2 cells after 72 h of incubation with extracts from titanium alloy samples, calculated in relation to the survival of cells after incubation with the negative control, was 107.94%.

**Figure 16:**
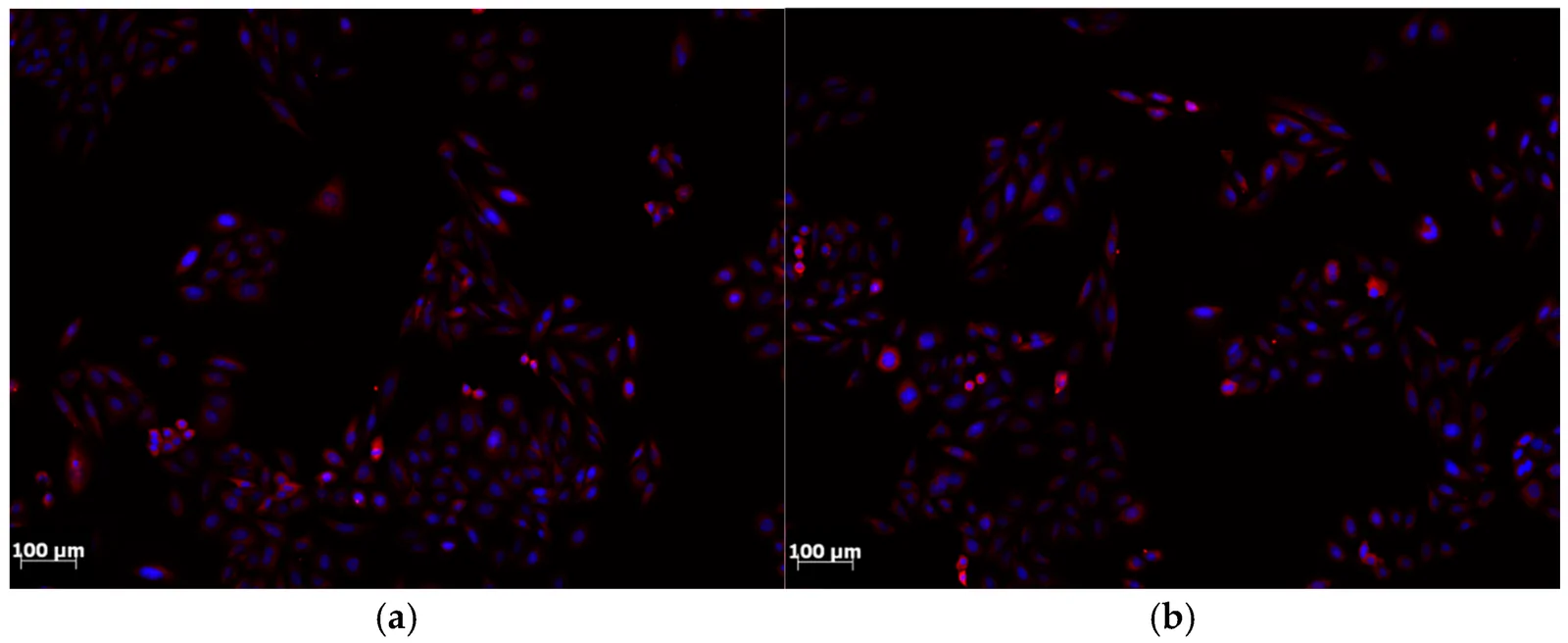
Fluorescence microscopy images of SaOS-2 cells: (**a**) control group of cells cultured under standard conditions without exposure to material extracts and (**b**) cells incubated with extracts from the Ti6Al7Nb alloy. The images demonstrate cell morphology, cytoskeleton organization and cellular response after exposure to the tested material [30].

The microscopic analysis showed proper cellular morphology and organization of the cytoskeleton. Numerous proliferating cells and single cells undergoing cellular death (apoptosis or necrosis) were observed. Single cells containing micronuclei-like structures were observed (Figure 16).

## 4. Discussion

The sample surfaces exhibited morphological features characteristic of sandblasting, while maintaining a high degree of surface homogeneity. The measured roughness parameters were Sa = 0.396 ± 0.084 μm and Ra = 0.343 ± 0.097 μm. The observed non-uniform surface coloration results from optical interference effects associated with variations in the thickness of the oxide layer formed during the anodic oxidation process (Figure 1 and Figure 2). Previous studies [31] have demonstrated that the surface topography of anodically oxidized specimens is strongly influenced by the pre-treatment procedures applied prior to oxidation. For samples oxidized at an applied voltage of 97 V, the oxide layer thickness was approximately 200 nm. The measured contact angle (Θ = 54°) indicates hydrophilic surface behavior. Based on the findings of Yamamoto et al., the wettability and surface roughness parameters of the Ti6Al7Nb alloy provide surface characteristics that are considered favorable for osteoconductive responses according to the previous literature [32].

Based on the diffractogram obtained by X-ray phase analysis of the examined Ti6Al7Nb alloy, αTi and βTi phases were identified in the surface layer. Moreover, it was found that there is stress in the layer and the obtained hardness profile is correlated with the XRD results, indicating strengthening in the subsurface zone. This may confirm that the sandblasting process of the titanium alloy under study strengthens its surface.

The analysis of the potentiodynamic curves did not show any breakdown potential, and the return curve is below the original curve, which demonstrates the resistance of Ti6Al7Nb alloy after anodic oxidation preceded by grinding and sandblasting to pitting corrosion. Moreover, the anodizing of titanium forms an oxide coating that is rather resistant to the attack of chloride ions present in PBS solution [33]. The potentiodynamic polarization results indicate improved electrochemical behavior of the anodically oxidized Ti6Al7Nb surface in PBS solution. However, the lower current density observed during the reverse scan should not be interpreted as unequivocal evidence of pitting corrosion resistance, since it may also result from repassivation of the passive oxide film. Additional electrochemical characterization using electrochemical impedance spectroscopy (EIS) would provide complementary information on the protective properties of the oxide layer and should be considered in future investigations. The authors’ extensive study [34] of the effect of treatment prior to anodic oxidation on the corrosion resistance of Ti6Al7Nb alloy showed that the layers obtained after sandblasting had the best resistance. The study analyzed the corrosion resistance of the alloy in the initial state as well as after 28-day exposure to Ringer’s solution. Complementary tests were carried out on the penetration of metal ions into the corrosive environment.

Analysis of the Ti profile (Figure 9) showed that the main metallic component of the alloy is not dominant on the native surface of the sample (0 min. etching time). There, titanium reached a concentration of about 3% similar to the surface concentration of Al. However, with the penetration depth into the layer, the Ti contribution increased exponentially achieving a stable value of about 15% for the Ar+ etching time in the range 25–130 min. Further depth penetration showed only a gradual increase of titanium content up to a concentration of about 20%. On the other hand, during the robust increase in the Ti content, the aluminum concentration decreased. After 25 min of etching, the Al concentration oscillated around 1–2%. Then, again grew to about 3.5% in depth corresponding to etching of 260–360 min. The niobium concentration was found to be minor compared to the other main alloy components. It remained constant at a value of 0.2% from the native surface to the depth obtained after 160 min etching. For longer etching, Nb content started to gradually increase, reaching ~2% for maximal etching time equal to 360 min.

Oxygen was revealed as the main component of the passive layer reaching a concentration of about 60–65% for a depth corresponding to the interval of 4–260 min (Figure 10) Ar+ etching. This value decreased slightly by a few percent in the next 100 min of ion interaction. Only the native surface was characterized by a lower oxygen content (about 45%) caused probably by the presence of excess carbon. The carbon content on the native surface reached almost 34%. This value drastically dropped after only a few minutes of ion etching (to ~10%) and then systematically decreased to about 2% for a maximum etching time of 360 min. The signal from silicon, like from oxygen, showed a lower concentration at the native surface (about 7%) than in the deeper layers of the alloy. It remains constant at around 15% for the entire etching time range.

During potentiostatic anodization, the applied electric field promotes the migration of Ti and O ions inward, leading to the formation and growth of a compact TiO_2_ layer. Simultaneously, alloying elements such as aluminum and niobium are incorporated into the passive film as their corresponding oxides. The final composition and thickness of the oxide layer are therefore governed by the dynamic balance between electrochemical oxide growth and field-assisted dissolution occurring at the oxide/electrolyte interface.

Phosphorus and calcium showed relatively the highest concentration on the native surface, around 3.5% and 2.5%, respectively (Figure 11). The contribution of these elements gradually disappeared to a depth corresponding to 30 min. of etching. The sulfur profile behaved similarly. It reached a maximum value of about 1% on the native surface and gradually vanished after 35 min. of etching. Sodium was residual at a concentration of about 0.4% on the surface and disappeared after 12 min. of etching. Phosphorus, calcium, sulfur and sodium are residues of the anodic oxidation process (they are present in the electrolyte used). Their presence in the passive layer may promote surface bioactivation by stimulating the formation of hydroxyapatite on the alloy surface [35].

The decomposition of the Ti 2p XPS peak (exemplary window spectrum: inset to Figure 12) revealed the existence of Ti components in metallic form and the 2nd, 3rd, and 4th oxidation states including the spin–orbit splitting of 2p transition (2p3/2 and 2p1/2) equaled 6.1 eV (Figure 12). The asymmetric Ti 2p3/2 component at position 453.1 eV was assigned to Ti(0): metal. The subsequent symmetric Ti 2p3/2 components at BE of 455.2 eV, 457.1 eV, and 458, 5 eV were assigned to Ti(II) in the form of TiO, Ti(III)–Ti_2_O_3_ and Ti(IV) as TiO_2_, respectively [34]. Generally, on the alloy native surface, there was 90% titanium in the form of TiO_2_. This situation changed drastically within the first minutes of etching when the amount of TiO_2_ dropped to about 35% of the total titanium content. There, the Ti_2_O_3_ and TiO fractions also appeared in 40% and 25% of Ti, respectively. Above 150 min. of etching, an increase in the contribution of titanium in metallic form became noticeable. It reached a value of about 60% of the Ti signal in the 260–360 min. etching range. For this period, TiO and Ti_2_O_3_ showed a signal of ~15% Ti, while TiO_2_ had a contribution of ~6%.

Peak Al 2p decomposition revealed the existence of Al components in metallic form (with spin–orbit splitting equaled 0.42 eV [35]) and in 3rd oxidation states (Figure 13). The asymmetric Al 2p3/2 component located at 71.0 eV was attributed to Al(0): metal, whereas the symmetric, wide one at 74.0 eV was assigned to Al(III): Al_2_O_3_ [36,37]. From the alloy’s native surface to the depth corresponding to ~150 min. of etching, the aluminum exists only in the form of Al_2_O_3_. Then, an abrupt decrease in oxide content was observed reaching a level of ~50% of the total Al signal after 200 min. The next 50% was related to Al in the metallic form. The situation was stable up to 360 min of Ar+ sputtering.

Peak Nb 3d decomposition showed the existence of Nb components in metallic form and the 2nd and 5th oxidation states with the spin–orbit splitting of 3d transition (3d5/2 and 3d3/2) equaled 2.7 eV (Figure 14) [37]. The asymmetric Nb 3d5/2 component located at 202.4 eV was attributed to Nb(0): metal [38]. The next symmetric Nb 3d5/2 components at 203.8 eV and 207.9 eV were assigned to Nb(II) in the form of NbO and/or NbC, Nb(V)–Nb_2_O_5_, correspondingly [38]. On the alloy native surface and for the very first minute of argon sputtering the niobium signal was on noise level likely due to surface contaminations. For more than 8 min. of etching, the phases of NbO/NbC and Nb_2_O_5_ appeared and their amount oscillated around 50% of total Nb content. After 60 min. of Ar treatment, the metallic component appeared. It started to grow up gradually reaching ~70% after 145 min. At the same time, the niobium oxide components’ contribution continuously decreased. Finally, the NbO/NbC input to the Nb signal disappeared after 215 min, and the Nb_2_O_5_ signal after 315 min of etching.

The decomposition of the O 1s XPS peak manifested the appearance of four oxygen components (Figure 15). The O1 positioned at 530.3 eV was attributed to metal oxides, and the O2 at BE = 531.6 eV was assigned to metal oxides’ defects [39,40,41,42]. The next one (O3), located at 533.0 eV, originated from carbon-related species C-O, C=O and probably from SiO_2_ oxide [12,13]. The Si2p signal observed after sandblasting is attributed to trace oxidized silicon species, most likely residual SiO_2_ [43] originating from the abrasive medium. Such contamination is not expected to adversely affect the biocompatibility of Ti6Al7Nb; however, it may slightly modify the surface physicochemical properties [44]. The fourth component (O4) at 534.1 eV was related to OH/water bonds [44,45,46]. On alloy native surface dominates signals from O2 and O3 species (both ~32% of total oxygen content). It corroborated with a significant amount of carbon detected on the alloy surface. There, metal oxides, as well as water phases (O1 and O4, respectively), were evenly on the level of ~18%. But, after 4 min of etching, metal oxide contribution increased suddenly up to ~50% and stayed stable till 85 min of Ar+ sputtering. Then, O1 tended to fall slowly reaching a final value of ~40% for maximum etching time. A similar effect of oxide content decrease within the corresponding alloy depth was observed for titanium, aluminum and niobium (Figure 12, Figure 13 and Figure 14). The other oxygen components, O2, O3 and O4, within the argon sputtering period of 85–360 min remained stable at the level of approximately 25%, 20% and 15%, respectively.

Tests of the samples showed that the passive layer consists mainly of oxides of alloying elements Ti, Al, Nb, the contribution of which decreases over time as the contribution of metallic elements increases. The XPS depth profiles indicate that titanium was predominantly present in the form of TiO_2_ within the surface layer, confirming that anodic oxidation resulted in the formation of a stable oxide film. The gradual changes in elemental concentrations with increasing sputtering depth reflect the transition from the oxide layer to the metallic substrate. The presence of aluminum and niobium oxides within the passive film is consistent with the alloy composition and may contribute to the stability and protective properties of the oxide layer.

Biocompatibility tests allowed to conclude that the analyzed titanium alloy surface does not cause any cytotoxic effect. Moreover, cells of the SaOS-2 line, both in the control group and after incubation with Ti6Al7Nb alloy extracts, did not show any changes in their morphology and organization (Figure 16). The biological evaluation performed in this study was limited to the extract cytotoxicity assay in accordance with ISO 10993-5 and ISO 10993-12, which represents an initial screening method for assessing the biological safety of biomaterials. While the absence of cytotoxic effects confirms that no harmful substances were released from the modified surface under the applied test conditions, this approach does not provide direct information on cell–surface interactions, such as cell adhesion, spreading, proliferation, or osteogenic differentiation. Therefore, further studies involving direct-contact cell assays and more advanced biological evaluations are required to comprehensively assess the biological performance and osteoconductive potential of the anodically oxidized Ti6Al7Nb surface.

Overall, the obtained results demonstrate a clear relationship between the anodizing process, the chemical composition of the oxide layer, and the resulting functional properties of the Ti6Al7Nb alloy. The formation of a homogeneous TiO_2_-rich passive layer preserved the favorable roughness generated by sandblasting, increased surface wettability, and improved corrosion behavior without adversely affecting cytocompatibility. These findings indicate that the physicochemical modifications introduced by anodic oxidation play a key role in determining the biological and electrochemical performance of the modified surface.

## 5. Conclusions

1. The present study provided a comprehensive physicochemical, electrochemical, and biological characterization of the Ti6Al7Nb alloy surface after the combined process of sandblasting and anodic oxidation. XPS depth profiling confirmed that the anodically formed passive layer consisted predominantly of TiO_2_, together with oxides of alloying elements, indicating the formation of a stable oxide film.
2. The obtained surface exhibited favorable physicochemical characteristics, including moderate roughness, hydrophilic behavior, and the presence of phosphorus and calcium species. These surface features, together with the TiO_2_-rich passive layer, were associated with improved corrosion behavior in PBS solution and are considered beneficial for interactions with the biological environment.
3. The indirect cytotoxicity assay demonstrated that extracts from the modified surface did not induce cytotoxic effects in SaOS-2 cells, confirming the preliminary in vitro cytocompatibility of the investigated surface.
4. The results indicate that the proposed surface modification represents a promising approach for improving the functional properties of Ti6Al7Nb alloy intended for biomedical applications. However, the present study constitutes a preliminary in vitro evaluation. Further investigations involving direct cell–surface interaction assays, osteogenic activity, long-term electrochemical characterization, and in vivo studies are required to fully assess the biological performance and clinical potential of the modified surface.

## Figures and Tables

**Table 1:** Selected structural parameters determined using Rietveld refinement [30].

Lattice Parameters, ICDD, Å	Lattice Parameters, Calculated, Å	Crystallite Size, nm	Lattice Strain, %	Compressive Stress, MPa
a = 2.951, c = 4.683	a = 2.950 c = 4.695	30	0.89	−544(21)

## Data Availability

The original contributions presented in this study are included in the article. Further inquiries can be directed to the corresponding authors.
